# Safety of human papillomavirus 6, 11, 16 and 18 (recombinant): systematic review and meta-analysis

**DOI:** 10.1016/j.rpped.2015.02.006

**Published:** 2015

**Authors:** Pedro Luiz Spinelli Coelho, Gustavo Lacerda da Silva Calestini, Fernando Salgueiro Alvo, Jefferson Michel de Moura Freitas, Paula Marcela Vilela Castro, Tulio Konstantyner

**Affiliations:** a Centro Universitário Lusíada, Santos, SP, Brazil; b Universidade de Santo Amaro, São Paulo, SP, Brazil; c London School of Hygiene & Tropical Medicine, Londres, England

**Keywords:** Papillomavirus vaccines, Adverse effects, Adolescent, Meta-analysis, Safety

## Abstract

**Objective:**

To identify and quantify the adverse effects associated with the recombinant human papillomavirus (types 6, 11, 16 and 18) vaccine in adolescents.

**Data source:**

Systematic review of randomized clinical trials from PubMed, SciELO and Lilacs databases. Articles investigating the safety of the vaccine in subjects under 18 years and comparing the recombinant human papillomavirus types 6, 11, 16 and 18 vaccine with a control group were included. Meta-analyses were performed for the outcomes of pain, erythema, swelling and fever, using clinical trials with maximum Jadad score.

**Data synthesis:**

Fourteen studies were included. The most common adverse effects related to the human papillomavirus vaccine were effects with no severity (pain, erythema, edema, and fever). Five studies were used for the meta-analyses: pain-risk difference (RD)=11% (*p*<0.001); edema-RD=8% (*p*<0.001); erythema-RD=5% (*p*<0.001); fever-RD=2% (*p*<0.003).

**Conclusions:**

The recombinant human papillomavirus types 6, 11, 16 and 18 vaccine was safe and well tolerated. The main adverse effects related to vaccination were pain, erythema, edema and fever. The low frequency of severe adverse effects encourages the administration of the vaccine in the population at risk.

## Introduction

Cervical cancer is the second most common type of cancer that affects women worldwide, with an incidence of approximately 500,000 cases and 270,000 deaths each year.^1^^,^ 2 The disease is often detected at advanced stages due to the lower efficiency of screening strategies in the initial stage and treatment options that are not always effective.^3^^-^ 6 At least 80% of deaths from cervical cancer occur in developing countries, most of them in the poorest regions of the world, such as Southern Asia, Sub-Saharan Africa and parts of Latin America. In those areas, which receive only 5% of the resources for cancer in the world, cervical involvement is responsible for 15% of all cancer deaths.^7^

Infection by human papillomavirus (HPV) is a common occurrence, and the probability of acquiring it throughout an individual's lifetime is higher than 50%.^8^ Approximately 35-40 types of HPV can infect the genital epithelium. The infection may be transient and not clinically detectable, but can also cause genital warts and a variety of pre-malignant and malignant anogenital lesions in both genders.^9^^-^ 14 Studies show that the peak incidence of HPV infection occurs 5-10 years after the first sexual intercourse (between 15 and 25 years old),^15^^-^ 19 and infection persistence by an oncogenic HPV type is crucial in the pathogenesis of cervical cancer.^2^^,^ 20 ^-^ 22 Thus, it becomes possible to prevent the disease onset through vaccination before the start of sexual activity.^19^^,^ 23 ^-^ 26

The currently available vaccines against HPV differ in the number of genotypes, in the way they are manufactured and the adjuvant they contain. Both vaccines currently available for use, bivalent and quadrivalent, are highly immunogenic and prevent the primary infection against HPV genotypes and CIN 2/3 adenocarcinoma (CIN - cervical intraepithelial neoplasia, which refers to squamous epithelial lesions in the lower genital tract, which are precursors of invasive cancer, presenting as tissue impairment, from cytoplasmic alterations to severe dysplasia). Studies indicate a very similar safety profile for severe and mild adverse effects for each one of the vaccines.^27^^,^ 28

The introduction of new vaccines requires safety studies. Concerns about the adverse effects is considered a barrier to vaccination and one of the reasons for low adherence to the recommendations for human papillomavirus quadrivalent (types 6, 11, 16, 18) recombinant vaccine administration.^29^^,^ 30 The opinion of health professionals regarding its safety is yet to be unanimous. Several debates have been carried out with persistent controversies about the advantages and disadvantages of its use. Therefore, the knowledge of the possible local and systemic adverse effects can subsidize adherence strategies and guide health care actions for the population at risk.

Therefore, the objective of this study is to identify and quantify the adverse effects associated with the administration of the human papillomavirus quadrivalent (types 6, 11, 16, 18) recombinant vaccine, as a tool to determine the safety of its use in adolescents.

## Method

A search for publications was carried out in April 2014, in the National Center for Biotechnology Information Advances Science and Health - US National Library of Medicine - National Institutes of Health - PubMed electronic databases, with no restrictions regarding date and language of publication. Additionally, a search was performed in the LILACS and SciELO databases using the descriptor "Papillomavirus Vaccines", followed by a manual search for randomized controlled trials (RCTs). In the first stage of article selection, the Decs/Mesh health descriptor "papillomavirus vaccines/adverse effects" was used. The study design filter "RCTs" was added to the obtained results. Subsequently, the identified articles were analyzed by reading the titles and abstracts.

At this stage, the exclusion criteria of the articles were: concurrent use of HPV vaccine with other vaccines; use of the vaccine in patients who already had cervical diseases; patients positive for HIV; patients who had already received the HPV vaccine; studies on the acceptance among family members of the administration of HPV vaccine in adolescents; studies carried out exclusively with patients aged >18 years; repeated studies and systematic reviews.

All other studies were read in full, analyzed by five independent investigators and classified according to the Jadad score.^31^ In this classification, one point was attributed for each of the following items: description of article as randomized trial and description of article as double blind; one additional point for each of the articles of which method was described, and in case it was appropriate; the point for randomization and blinding was subtracted if the method used for those was inappropriate; and an additional point for description of losses.

**Figure 1:**
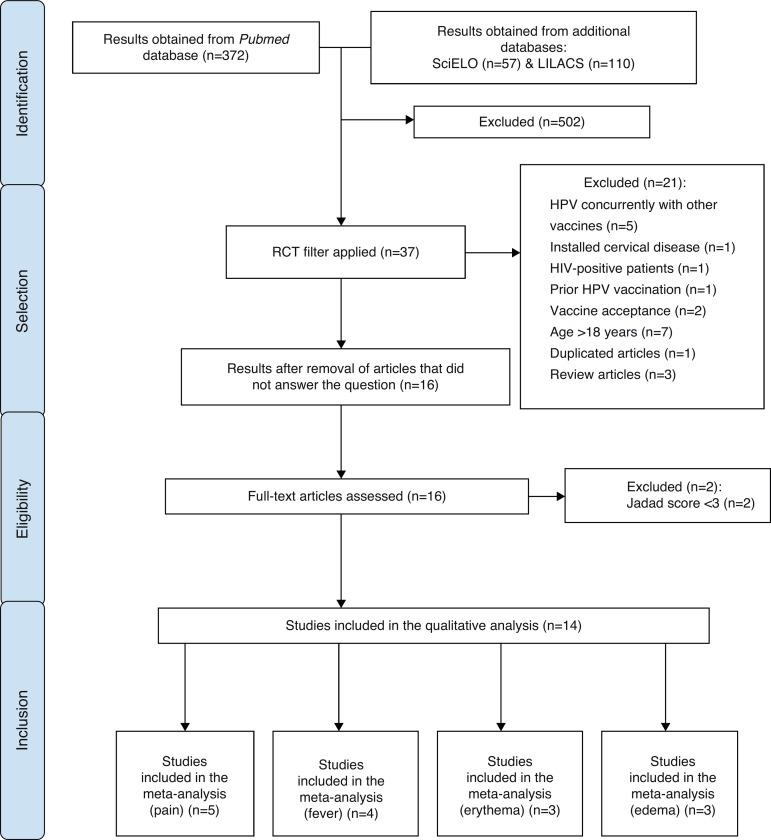
Article selection process.

After this step, the researchers met at a panel discussion on eligibility criteria. Therefore, articles that evaluated the safety of HPV bivalent and quadrivalent vaccines, in both genders, with Jadad score^31^≥3 were included. The process of article selection is shown in Fig. 1.

Initially, all adverse effects identified in the selected RCTs were listed. The selected articles were described according to the results shown in each of the RCTs, disclosing prevalence data and characteristics of the identified adverse effects. Subsequently, four meta-analyses of four different outcomes evaluated in RCTs (pain, edema, erythema, fever) were performed, which included articles with Jadad score = 5^31^ and compared their occurrence between the group that received the HPV quadrivalent vaccine and the placebo group.

The statistical package Review Manager 5.2 software was used. The results were expressed as risk difference (RD) with fixed confidence interval of 95% and a statistical significance level with a maximum *p*=0.05 (5%). Heterogeneity was calculated using the statistical Mantel-Haenszel chi-square test, and expressed as *I* ^2^, and those with value >50% (*I* ^2^>50%) were considered heterogeneous. Asymmetries were represented in the funnel plot.

## Results

A total of 14 RCTs were included in this study, none of them from the SciELO or LILACS databases. Table 1 shows the adverse effects associated with the administration of the human papillomavirus quadrivalent (types 6, 11, 16, 18) recombinant vaccine, without necessarily establishing a causal link between the vaccine and the effects, indicating how many of these studies identified them and the variations between prevalence rates.

**Table 1:** Frequency of identification (FI) and prevalence interval (PI) of adverse effects associated with the HPV vaccine and identified in 14 selected randomized controlled trials.

Adverse effects	FI	PI (%)
*Local*		
Local pain	13	20.2–88.4
Edema	12	3.0–31.0
Erythema	11	1.0–41.0
Pruritus	3	1.1–4.1

*Systemic*		
Fever	12	2.7–23.5
Fatigue	9	0.3–38.8
Headache	12	5.3–35.2
Cough	2	3.6
Arthralgia	7	5.5–13.4
Myalgia	8	3.6–33.9
Sore throat	3	0.4–4.0
Nausea	2	0.8–2.6
Chills	1	–
Abdominal pain	2	0.3
Diarrhea and/or vomiting	12	0.5–19.3
Acne	1	0.8
Dizziness and vertigo	3	1.0

*Diseases*		
Psychiatric	2	1.7–2.2
Blood and lymphatic system	1	0.4
Cardiovascular	3	0.1–0.6
Ear and labyrinth	1	1.2
Nervous system	4	1.3–33.7
Ocular	1	1.0
Immunological/allergies	3	0.6–2.6
Metabolic and nutritional	1	0.8
Infectious	1	20.7
Renal and urinary	3	0.4–1.4
Reproductive and breasts	1	8.4
Respiratory, thoracic or mediastinal	1	0.9–7.7
Skin and subcutaneous tissue	3	4.2
Musculoskeletal and of connective tissue	5	1.1–9.1
Mastoiditis	1	–
Upper airways infections	11	0.1–6.0
Diabetes mellitus	2	–
Renal failure	1	–
Poisoning, trauma	2	–
Intoxication	1	1.7
Appendicitis	1	–
Rash/hives	7	0.8–6.6
Hypothyroidism	1	0.4
Malaria	1	–

*Perinatal*		
Dysfunctional uterine bleeding	1	–
Prematurity	2	–
Congenital anomaly	1	2.3
Late fetal death	1	–
Miscarriage	1	22.2

FI, frequency of identification; PI, prevalence interval.

Table 2 describes the characteristics of the selected studies during the search process,^23^^,^ 32 ^-^ 43 including authorship, year of publication, classification according to the Jadad score,^31^ methodology used and the adverse effects that were statistically related to the vaccine.

**Table 2:** Characteristics of 14 randomized clinical trials selected by the used search criteria.

Author (year/JADAD)	Method			Effects associated with the vaccine
	Design	Intervention	Gender (age range)	
Moreira et al. ^[^ 34 ^]^ (2011/5)	RCT, DB, PC	HPV QV (*n*=1945) *vs.*Placebo (*n*=1950)	M (16–26 yrs)	Local pain
Kim et al. ^[^ 23 ^]^ (2010/5)	RCT	HPV DV (*n*=474) *vs.*hepatitis A vaccine (*n*=483)	F (10–14 yrs)	Local pain, erythema, edema and myalgia
Sow et al. ^[^ 35 ^]^ (2013/5)	RCT IIIb, DB, PC, MULTI (aluminum hydroxide)	HPV DV (*n*=1298) *vs.*Placebo (*n*=643)	F (10–25 yrs)	Local pain
Reisinger et al. ^[^ 36 ^]^ (2007/5)	RCT, DB, PC	HPV QV (*n*=1165) *vs.*Placebo (*n*=584)	M and F (1:1) (9–15 yrs)	Local pain, erythema and edema
Szarewski et al. ^[^ 37 ^]^ (2012/5)	RCT, DB	HPV DV (*n*=9319) *vs.*hepatitis A vaccine (*n*=9325)	F exposed to HPV (15–25 yrs)	Local pain, erythema and edema
Petäjä et al. ^[^ 38 ^]^ (2009/3)	RCT, phase I/II, blind observer	HPV DV (*n*=181) *vs.*hepatitis B vaccine (*n*=89)	M (10–18 yrs)	Local pain, edema and myalgia
FUTURE II ^[^ 33 ^]^ (2007/5)	RCT, DB, PC	HPV QV (*n*=448) *vs.*Placebo (*n*=447)	F (15–26 yrs)	Local pain, seasonal allergies
Medina et al. ^[^ 39 ^]^ (2010/3)	RCT, MULTI	HPV DV (*n*=1017) *vs.*hepatitis A vaccine (*n*=1010)	F (10–14 yrs)	Local pain, erythema, edema, headache, myalgia, fatigue, hives and syncope
Romanowski et al. ^[^ 40 ^]^ (2011/3)	RCT phase I/II, PB; 2 *vs.* 3 doses of divalent HPV	2 Doses FOR20/20 M0/6 (*n*=240); 2 doses FOR40/40 M0/6 (*n*=241); 2 doses FOR40/40 M0/2 (*n*=240); 3 doses FOR20/20 0/1/6 (*n*=239)	F (9–25 yrs)	–
Garland et al. ^[^ 32 ^]^ (2007/5)	RCT, DB, PC, MULTI	HPV QV (*n*=2673) *vs.*Placebo (*n*=2672)	F (16–24 yrs)	Local pain, erythema, edema and local pruritus, fever >38.9 °C, bronchospasm
Petäjä et al. ^[^ 41 ^]^ (2011/3)	RCT phase III, MULTI	HPV DV (*n*=616)	F (10–25 yrs)	–
Esposito et al. ^[^ 42 ^]^ (2011/3)	RCT, standard schedule (0/1/6 m) *vs.*alternative (0/1/12 m)	HPV DV standard (*n*=1195) *vs.* HPV DV alternative (*n*=1188)	F (15–25 yrs)	–
Kang et al. ^[^ 43 ^]^ (2008/5)	RCT, DB, PC	HPV QV (*n*=117) *vs.*Placebo (*n*=59)	F (9–23 yrs)	Local pain, erythema, edema and fever
Li et al. ^[^ 44 ^]^ (2011/3)	RCT, DB, PC	HPV QV (*n*=302) *vs.*Placebo (*n*=298)	M (9–15 yrs) and F (9–45 yrs)	Local pain, edema and pruritus

RCT, randomized controlled trial; DB, double-blind; PB, partially blind; PC, placebo-controlled; MULTI, multicenter; HPV, human papillomavirus; M, male; F, female; FOR, formulation; m, month; DV, divalent; QV, quadrivalent; yrs., years.

Among the analyzed studies, there was only one case of severe adverse event related to the vaccine, which was bronchospasm.^32^ The others showed no reports of vaccine-related severe adverse effects or deaths. The incidence of adverse effects was higher after the first dose of the vaccine schedule, with a reduction in their occurrence at subsequent doses.^23^^,^ 33 ^-^ 36 ^,^ 43

The selection of outcomes for the meta-analyses was carried out according to the frequency of appearance of adverse effects assessed in the selected publications, comparing their occurrence between vaccinated and unvaccinated subjects against HPV, emphasizing the local effects (pain, erythema and edema) and, as systemic effect, fever.

**Figure 2:**
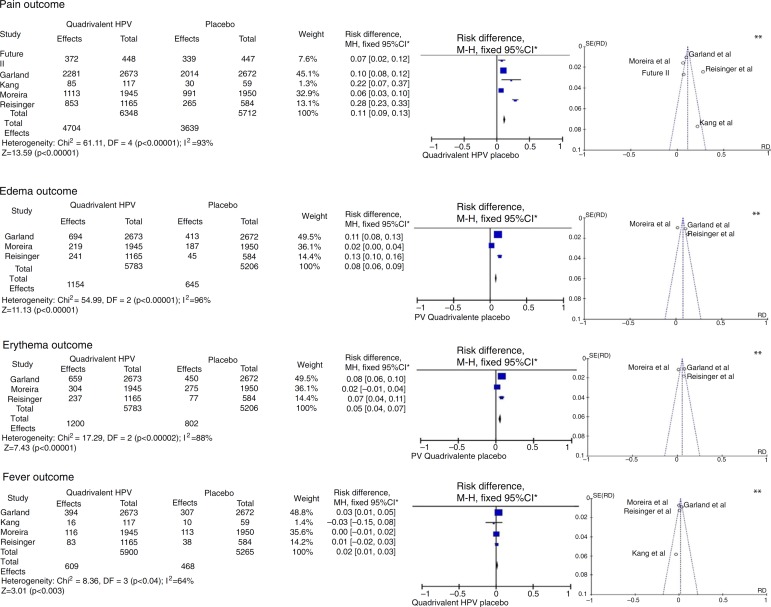
Meta-analysis of outcomes "pain", "edema", "erythema" and "fever" from the results provided by the selected RCTs. *M-H, Mantel-Haenszel; CI, confidence interval; in the forest plots, the horizontal axis represents the CI of risk difference. The points represent the risk difference in each study. The dots located to the right of the median line indicate higher incidence of the outcome in the group that received the vaccine; the size of the dots represents the relative weight of each study in the final outcome. The diamond indicates the final outcome of the meta-analysis; ** funnel plots (horizontal axis = magnitude of the effect; vertical axis = sample size) illustrate the heterogeneity between studies.

This analysis was performed with five studies with a Jadad score^31^ of 5,^32^^-^ 34 ^,^ 36 ^,^ 43 without gender or age group restriction for the quadrivalent vaccine, with four of them being multicenter studies^32^^-^ 34 ^,^ 36 and one having been carried out in South Korea.^43^ The meta-analysis results for the "pain", "edema", "erythema" and "fever" outcomes are shown in Fig. 2.

For the "pain" outcome, the meta-analysis assessed five RCTs, totaling 12,060 participants, 6348 vaccinated for HPV and 5712 placebo-controlled. Of the vaccinated ones, 4704 had pain at the injection site, while only 3639 reported the same outcome with the placebo, resulting in an RD=11% (95%CI: 9-13%; *I* ^2^=93%), therefore being significantly more common after the administration of the vaccine (*p*<0.001).

The "edema" outcome was analyzed in three multicenter articles.^32^^,^ 34 ^,^ 36 These included 5783 patients, of which 1154 developed edema at the injection site in the group vaccinated for HPV, and 645 of 5206 in the placebo group, resulting in an RD=8% (95%CI: 6%-9%; *I* ^2^=96%) for this outcome in favor of the vaccinated group (*p*<0.001).

The meta-analysis of the "erythema" outcome included the same articles used for edema. Of all patients vaccinated for HPV, 1200 developed erythema at the injection site, while 802 had erythema in the placebo group, with an RD=5% (95%CI: 4-7%, *p*<0.001, *I* ^2^=88%).

In turn, the meta-analysis of the "fever" outcome involved four articles. Of the 5900 vaccinated patients, 609 developed fever, while 468 of the 5265 in the placebo group had the same outcome. The RD=2% (95%CI 1-3%, *I* ^2^=64%) was significant (*p*<0.003).

## Discussion

Considering that the Ministry of Health of Brazil has only recently incorporated this vaccine into the immunization schedule of female adolescents, and also the future possibility of expanding its target audience, the study of the safety profile of the human papillomavirus quadrivalent (types 6, 11, 16, 18) recombinant vaccine is crucial. Moreover, considering its good efficacy demonstrated in several studies,^32^^,^ 33 ^,^ 39 ^,^ 41 the clear association between cervical cancer and chronic HPV infection and the need for the administration of more than one dose to meet the purpose, the knowledge of possible adverse reactions is important to ensure adherence to the proposed vaccination schedule and, consequently, the success of this strategy to prevent this infectious disease as well as cervical cancer.

In this context, the clinical trials selected for this study suggest the existence of several local and systemic adverse effects, severe or not, chronic disease onset during the study period and new relevant medical conditions reported by the affected patients. However, most of these clinical phenomena cannot be defined as adverse effects associated with the administration of the human papillomavirus quadrivalent (types 6, 11, 16, 18) recombinant vaccine, for their occurrence has not been compared to control groups, and the causal association has not been established.

In two articles, different immunization schedules were tested and compared and no significant differences were found regarding the occurrence of pain or other adverse effects between the groups.^40^^,^ 42 The remaining 12 articles compared groups receiving the human papillomavirus quadrivalent (types 6, 11, 16, 18) recombinant vaccine and control groups. For these 12 studies, local adverse effects are noteworthy, as they were the most frequently associated with HPV vaccination, when compared to control groups. In all of these articles, there is at least one adverse effect statistically associated with the vaccination, which shows the importance of safety analysis. Among the effects were pain, erythema, edema and pruritus.

Pain was identified in 11 of the 12 articles and this was the most common adverse effect, always associated with human papillomavirus quadrivalent (types 6, 11, 16, 18) recombinant vaccine when compared to the placebo group,^32^^-^ 36 ^,^ 43 ^,^ 44 to the control group with hepatitis A vaccine 23 ^,^ 37 ^,^ 39 or hepatitis B vaccine.^38^ Edema was present in ten articles. Of these, eight identified edema as an effect that was directly related to HPV vaccination. Erythema was described in nine articles, but only six showed a direct association with vaccination. Pruritus was an adverse effect that was rarely found among the assessed articles, being present in only three of them.

Given the magnitude of the identified local adverse effects, the performance of the meta-analysis aimed not only to confirm, but also to quantify them, using statistical methods that allow a joint assessment of trials investigating the association of adverse effects with the human papillomavirus quadrivalent (types 6, 11, 16, 18) recombinant vaccine. The meta-analyses performed in this study showed a higher probability of vaccinated individuals to develop local effects, with significant difference of risk, especially regarding local pain, and to a lesser extent, erythema and edema.

Moreira et al.^34^ suggest that the adjuvant AS04 may be implicated in a higher incidence of local adverse effects; however, its role is of utmost importance to enhance the vaccine immunogenicity. As this finding was exclusively found in the trial carried out by these authors, further studies are required to determine the causal association, which is likely to change the use of this adjuvant in the vaccine manufacturing process.

Among the wide variety of systemic adverse effects identified in this review, few were actually related to immunization, such as fever and myalgia. Ten of the 12 articles listed fever as an adverse effect, but only two of them were related to vaccination when compared to controls. The risk of fever after the administration of the human papillomavirus quadrivalent (types 6, 11, 16, 18) recombinant vaccine observed in the meta-analysis was greater than that in the placebo group. This fact should be a warning to health care professionals who recommend care after prescribing the vaccine to their patients. However, it is noteworthy that although this risk difference is significant, it is not sufficient to contraindicate its use, based on the benefit of protection against viral infection and cervical cancer.

Other mentioned systemic effects, such as headache, rash, myalgia, hives, syncope, fatigue,^39^ and allergies^33^ were systemic adverse effects also related to vaccination, but were not included in the meta-analyses, as they were not reproduced in other studies. The other systemic adverse effects, which differed between articles, were unrelated to the vaccination and showed no difference between the study groups regarding their occurrence, positively contributing to its safety profile. Some of them did not have an explicitly reported frequency of occurrence, making it difficult to analyze them. This observation leads us to consider the need for additional long-term studies, and with larger sample sizes, so that rare outcomes can be assessed.^32^^,^ 39

Although they are frequent, more severe adverse effects related to vaccination were not described and, for the most part, they were not a cause of losses in the completion of the multiple vaccination schedule. It was also observed that the incidence of adverse effects is higher after the first dose of the schedule, with decreasing occurrence in subsequent doses.^23^^,^ 34 ^-^ 36 ^,^ 43

Considering the prevalence of HPV infection in adolescence and the incidence and morbimortality of cervical cancer, the risk/benefit ratio of the vaccine shows to be completely acceptable, confirming its good tolerability and safety proposed by other authors,^23^^,^ 32 ^-^ 36 ^,^ 39 ^-^ 44 which contributes to the effectiveness of public health policies.

Despite the identification of effects in observational studies, which can contribute to the construction of knowledge, the present study chose the exclusive selection of RCTs with comparison to a control group, especially those using placebo, as studies with this design allow establishing a clear association of adverse effects with vaccination and rule out the undesirable effect of confounding factors, often present in observational designs. Thus, the findings here are the result of the knowledge generated by the maximum level of scientific evidence available in the literature. However, it is noteworthy that the RCTs that evaluate the safety and reactogenicity of the vaccine include a limited number of subjects when compared with the general population of individuals eligible for vaccination. This characteristic restricts the identification of rare or unknown adverse effects. Therefore, the results shown herein should be interpreted with caution.

This study did not differentiate between genders, because although the mass vaccination was recommended for female adolescents, the male population is also susceptible to infection and diseases caused by HPV. Additionally, the male population can play a key role in disease transmission, and one should consider the possibility of this being a possible target of vaccination campaigns in the future. Reisinger et al.^36^ suggest that the female population reports more adverse effects than the male population, but no formal comparison was made between genders for this finding. Moreover, the selected articles showed great variability in the assessed age group, including schoolchildren, adolescents, young adults and adults.

This fact also contributed to differences between the study populations and, possibly, to the external validity of the obtained results. Therefore, the selection of articles could not limit the age group, as there was no standard methodology between studies regarding the age of the study population.

The meta-analyses showed moderate and high heterogeneity values (*I* ^2^ > 50%), which reduces the degree of confidence in the results shown here. Overall, the results followed the trend of a large sample population study.^32^ Some variables may be attributed as the cause of the heterogeneity, especially age, gender and sample size, suggesting that the outcomes studied in the meta-analyses cannot be dependent on the vaccine only, but also on the abovementioned factors. On the other hand, the confidence intervals of the calculated risk differences were narrow, due to the similarity of the results identified in each trial selected to comprise the meta-analyses, reinforcing the plausibility of the observed magnitude effect.

Another methodological divergence occurred regarding the control groups of each study. Some studies performed comparisons with a control group that used placebo^32^^-^ 36 ^,^ 43 ^,^ 44 or control with hepatitis A^23^^,^ 37 ^,^ 39 or hepatitis B vaccine.^38^These articles were maintained in this study, because hepatitis A and B vaccines have a well-established use, with a well-defined safety profile, and can be a good reference. However, the meta-analysis used only articles that compared the human papillomavirus quadrivalent (types 6, 11, 16, 18) recombinant vaccine with placebo, so that the sample would be as homogeneous as possible. Moreover, information on the intensity of the assessed adverse effects has not been demonstrated in all the selected studies, which might impair the evaluation of the effect severity. Our study, therefore, did not consider differences in severity when all outcomes were analyzed together.

Although it was not the focus of this study, it is noteworthy that, when the bivalent and quadrivalent vaccines are compared, the results suggest that both have similar safety profile and adverse effects, with a predominance of local effects. However, this finding can only be established by performing further studies, of which objective is to compare the occurrence of adverse effects between the vaccines.

In this context, the results shown here suggest that the use of human papillomavirus quadrivalent (types 6, 11, 16, 18) recombinant vaccine is potentially safe and well tolerated. The main adverse effects related to vaccination are local effects, such as pain, erythema and edema. As for systemic effects, fever was associated with vaccination. Both groups of adverse effects were not considered severe. Finally, it is concluded that the high immunogenicity and safety profile of the human papillomavirus quadrivalent (types 6, 11, 16, 18) recombinant vaccine determine that its use has an advantageous risk/benefit ratio and is a favorable strategy to prevent this viral infection as well as cervical cancer, which supports the persistent encouragement from health professionals to provide HPV vaccination to the risk population.
