# Supplementary material for: Safety of human papillomavirus 6, 11, 16 and 18 (recombinant): systematic review and meta-analysis
Source: Rev Paul Pediatr. 2015 Oct-Dec;33(4):474–82. doi: 10.1016/j.rpped.2015.02.006 (PMC4685569; doi:10.1016/j.rpped.2015.02.006)
Supplement: Translation

# Translation: Segurança da vacina papillomavirus humano 6, 11, 16 e 18 (recombinante): revisão sistemática e metanálise

**Authors:** Pedro Luiz Spinelli Coelho, Gustavo Lacerda da Silva Calestini, Fernando Salgueiro Alvo, Jefferson Michel de Moura Freitas, Paula Marcela Vilela Castro, Tulio Konstantyner

**Affiliations:** a Centro Universitário Lusíada, Santos, SP, Brasil; b Universidade de Santo Amaro, São Paulo, SP, Brasil; c London School of Hygiene & Tropical Medicine, Londres, Inglaterra

**Keywords:** Vacinas contra papillomavirus, Efeitos adversos, Adolescente, Metanálise, Segurança

## Abstract

**Objetivo:**

Identificar e quantificar os efeitos adversos associados à vacina papillomavirus humano 6, 11, 16 e 18 (recombinante) em adolescentes.

**Fontes de dados:**

Revisão sistemática de ensaios clínicos randomizados nas bases de dados do PubMed, SciELO e Lilacs. Foram incluídos artigos que abordavam a segurança da vacina em menores de 18 anos e que comparavam a vacina papillomavirus humano 6, 11, 16 e 18 (recombinante) com grupo controle. Foram feitas metanálises para os desfechos de dor, eritema, edema e febre com o uso de ensaios clínicos com escore de Jadad máximo.

**Síntese dos dados:**

Foram incluídos 14 estudos. Os efeitos adversos mais comuns relacionados à vacina foram intercorrências sem gravidade (dor, eritema, edema e febre). Cinco estudos foram usados para as metanálises, incluindo os desfechos: Dor - Diferença de Risco (DR)=11% (*p*<0,001); Edema-DR=8% (*p*<0,001); Eritema-DR=5% (*p*<0,001); Febre-DR=2% (*p*<0,003).

**Conclusões:**

A vacina papillomavirus humano 6, 11, 16 e 18 (recombinante) mostrou-se segura e bem tolerada. Os principais efeitos adversos relacionados à vacinação foram dor, eritema, edema e febre. A baixa frequência de efeitos adversos graves encoraja a aplicação da vacina na população de risco.

## Introdução

O câncer de colo de útero é o segundo tipo de câncer que mais atinge mulheres em todo o mundo, com incidência de aproximadamente 500 mil casos e 270 mil mortes por ano.^1^^,^ 2 Frequentemente, a doença apresenta-se na forma avançada devido à menor eficiência de estratégias de rastreamento na fase inicial e às opções de tratamento nem sempre efetivas.^3^^-^ 6 Pelo menos 80% das mortes por câncer do colo do útero ocorrem em países em desenvolvimento. A maioria é proveniente das regiões mais pobres, como Sul da Ásia, África Subsaariana e partes da América Latina. Nessas áreas, que recebem apenas 5% dos recursos para câncer no mundo, o acometimento do colo do útero tem sido responsável por 15% de todas as mortes por câncer.^7^

A infecção pelo papillomavirus humano (HPV) é comum. A probabilidade de o indivíduo adquiri-la ao longo da vida excede 50%.^8^ Aproximadamente 35 a 40 tipos de HPV são capazes de infectar o epitélio genital. A infecção pode ser transitória e não detectável clinicamente, mas também pode ocasionar verrugas genitais e uma variedade de lesões anogenitais pré-malignas e malignas, em ambos os sexos.^9^^-^ 14Estudos mostram que o pico de incidência da infecção pelo HPV ocorre 5-10 anos após a primeira relação sexual (entre 15-25 anos)^15^^-^ 19 e a persistência de infecção por um tipo oncogênico do HPV é determinante na patogenia do câncer cervical.^2^^,^ 20 ^-^ 22 Dessa forma, passa a ser possível impedir que a doença se instale por meio da vacinação, antes do início da atividade sexual.^19^^,^ 23 ^-^ 26

As vacinas disponíveis atualmente contra o HPV diferem entre si no número de genótipos, na maneira como são fabricadas e no adjuvante que contêm. Ambas as vacinas disponíveis para uso, bivalente e quadrivalente, são altamente imunogênicas e previnem a infecção primária contra os genótipos de HPV e o adenocarcinoma NIC 2/3 (NIC - neoplasia intraepitelial cervical, refere-se a lesões escamosas do trato genital inferior precursoras do câncer invasivo e apresentam-se em níveis comprometimento tecidual, desde alterações citoplasmáticas a displasia grave). Estudos indicam um perfil de segurança muito semelhante para efeitos adversos graves e leves para cada uma das vacinas.^27^^,^ 28

A introdução de vacinas novas leva à necessidade de fazer estudos de segurança. A preocupação com os efeitos adversos é considerada uma barreira à vacinação e uma das causas da baixa aderência às recomendações da aplicação da vacina papillomavirus humano 6, 11, 16 e 18 (recombinante).^29^^,^ 30Ainda não é unânime a opinião de profissionais de saúde com relação à sua segurança. Diversos debates têm sido feitos com persistentes controvérsias sobre as vantagens e desvantagens de seu uso. Assim, o conhecimento dos possíveis efeitos adversos locais e sistêmicos pode subsidiar estratégias de adesão e orientar ações de cuidados para a população de risco.

Nesse contexto, o objetivo do presente estudo é identificar e quantificar os efeitos adversos associados à aplicação da vacina papillomavirus humano 6, 11, 16 e 18 (recombinante), como ferramenta para determinar a segurança de seu uso em adolescentes.

## Método

Foi feita uma busca de publicações na base de dados eletrônica National Center For Biotechnology Information Advances Science and Health - National Library of Medicine - National Institutes of Health - PubMed, sem restrição de data de publicação e linguagem, em abril de 2014. Adicionalmente, fez-se a busca nas bases de dados Lilacs e SciELO com o descritor "papillomavirus vaccines", seguida de busca manual por ensaios clínicos randomizados (ECRs). Na primeira etapa da seleção dos trabalhos usou-se o descritor de saúde Decs/Mesh "papillomavirus vaccines/adverse effects". O filtro de delineamento de estudo "ECRs" foi adicionado aos resultados obtidos. A seguir, os trabalhos resultantes foram analisados com a leitura dos títulos e dos resumos.

Nessa etapa, os critérios de exclusão dos artigos foram: aplicação da vacina do HPV concomitante a outras vacinas; uso da vacina em pacientes que já tinham doenças de cérvice uterino instaladas; pacientes com teste para o HIV reagente; pacientes já vacinados para o HPV; estudos sobre a aceitação entre parentes da aplicação da vacina do HPV em adolescentes; estudos exclusivamente com pacientes de idade superior a 18 anos; trabalhos repetidos e revisões sistemáticas.

Todos os trabalhos restantes foram lidos na íntegra, analisados por cinco investigadores independentes e classificados segundo o escore de Jadad.^31^ Nessa classificação, atribuiu-se um ponto para cada item a seguir: descrição do trabalho como randomizado e descrição do trabalho como duplo cego; um ponto adicional para cada um dos itens cujo método havia sido descrito e caso esse estivesse adequado; o ponto para randomização e cegamento era subtraído caso o método de se fazê-lo fosse inadequado; e ainda um ponto adicional para descrição de perdas.

Após essa etapa, os investigadores se reuniram em um painel de discussão de critérios de elegibilidade. Assim, foram incluídos artigos que avaliavam a segurança das vacinas bivalente e quadrivalente para o HPV, em ambos os sexos e com escore de Jadad^31^ ≥3. O processo de seleção dos artigos está descrito na Figura 1.

**Figura 1:** Processo de seleção dos artigos.

Inicialmente, todos os efeitos adversos identificados nos ECRs selecionados foram listados. Os artigos selecionados foram descritos de acordo com os resultados apresentados em cada um dos ECRs, como dados de prevalência e características dos efeitos adversos identificados. Posteriormente, fizeram-se quatro metanálises de quatro desfechos diferentes avaliados nos ECRs (dor, edema, eritema e febre), que incluíram artigos com escore de Jadad^31^ igual a cinco e compararam sua ocorrência entre grupo submetido à vacina quadrivalente do HPV e placebo.

O pacote estatístico usado foi o *software* Review Manager 5.2. Os resultados foram expressos em diferença de risco (DR) com intervalo de confiança fixo em 95% e nível de significância estatística com *p*-valor máximo de 0,05 (5%). A heterogeneidade foi calculada por meio do teste estatístico qui-quadrado de Mantel-Haenszel e expressa em I^2^. Foram considerados heterogêneos aqueles com valor acima de 50% (I^2^>50%). Assimetrias foram representadas no gráfico em funil (*funnel plot*)*.*

## Resultados

Foram incluídos no presente estudo 14 ECRs, nenhum deles proveniente da SciELO ou Lilacs. A Tabela 1 apresenta os efeitos adversos associados à aplicação da vacina papillomavirus humano 6, 11, 16 e 18 (recombinante), sem que necessariamente haja relação de causalidade entre a vacina e os efeitos, e aponta quantos desses estudos os identificaram e a variação entre as prevalências encontradas.

**Tabela 1:** Frequência de identificação e intervalo de prevalência de efeitos adversos associados com a aplicação da vacina contra o HPV e identificados nos 14 ensaios clínicos randomizados selecionados

Efeitos adversos	FI	IP (%)
*Locais*		
Dor local	13	20,2‐88,4
Edema	12	3,0‐31,0
Eritema	11	1,0‐41,0
Prurido	3	1,1‐4,1

*Sistêmicos*		
Febre	12	2,7‐23,5
Fadiga	9	0,3‐38,8
Cefaleia	12	5,3‐35,2
Tosse	2	3,6
Artralgia	7	5,5‐13,4
Mialgia	8	3,6‐33,9
Dor de garganta	3	0,4‐4,0
Náusea	2	0,8‐2,6
Calafrios	1	–
Dor abdominal	2	0,3
Diarreia e/ou vômitos	12	0,5‐19,3
Acne	1	0,8
Tontura e vertigem	3	1,0

*Doenças*		
Psiquiátricas	2	1,7‐2,2
Sangue e sistema linfático	1	0,4
Cardiovasculares	3	0,1‐0,6
Orelha e labirinto	1	1,2
Sistema nervoso	4	1,3‐33,7
Oculares	1	1,0
Imunológicas/alergia	3	0,6‐2,6
Metabólicas e nutricionais	1	0,8
Infecciosas	1	20,7
Renais e urinárias	3	0,4‐1,4
Reprodutivas e mamárias	1	8,4
Respiratórias, torácicas ou do mediastino	1	0,9‐7,7
Pele e tecido subcutâneo	3	4,2
Musculoesqueléticas e do tecido conectivo	5	1,1‐9,1
Mastoidite	1	–
Infecção das vias aéreas superiores	11	0,1‐6,0
Diabetes melito	2	–
Insuficiência renal	1	–
Envenenamento, traumas	2	–
Intoxicação	1	1,7
Apendicite	1	‐
Rash/Urticária	7	0,8‐6,6
Hipotireoidismo	1	0,4
Malária	1	–

*Perinatais*		
Sangramento uterino disfuncional	1	–
Prematuridade	2	–
Anomalia congênita	1	2,3
Morte fetal tardia	1	–
Aborto	1	22,2

FI, frequência de identificação; IP, intervalo de prevalência.

A Tabela 2 descreve as características dos estudos selecionados no processo de busca,^23^^,^ 32 ^-^ 43 que incluem a autoria, o ano de publicação, a classificação no escore Jadad,^31^ a metodologia usada e os efeitos adversos que foram relacionados estatisticamente à vacina.

**Tabela 2:** Características dos 14 ensaios clínicos randomizados selecionados pelos critérios de busca adotados

Autor (Ano/Jadad)	Método			Efeitos associados à vacina
	Delineamento	Intervenção	Gênero (faixa etária)	
Moreira et al.^34^ (2011/5)	ECR, DC, CP	HPV QV (n=1945) *vs.* Placebo (n=1950)	M (16‐26a)	Dor local
Kim et al.,^23^ (2010/5)	ECR	HPV BV (n=474) *vs.* Vacina hepatite A (n=483)	F (10‐14a)	Dor local, eritema, edema e mialgia
Sow et al.^35^ (2013/5)	ECR IIIb, DC, CP, MULTI (hidróxido de alumínio)	HPV BV (n=1298) *vs.* Placebo (n=643)	F (10‐25a)	Dor local
Reisinger et al.^36^ (2007/5)	ECR, DC, CP	HPV QV (n=1165) *vs.* Placebo (n=584)	M e F (1:1) (9‐15a)	Dor local, eritema e edema
Szarewski et al.^37^ (2012/5)	ECR, DC	HPV BV (n=9319) *vs.* Vacina hepatite A (n=9325)	F expostos HPV (15‐25a)	Dor local, eritema e edema
Petäjä et al.^38^ (2009/3)	ECR, fase I/II, observador cego	HPV BV (n=181) *vs.* Vacina hepatite B (n=89)	M (10‐18a)	Dor local, edema e mialgia
FUTURE II^33^ (2007/5)	ECR, DC, CP	HPV QV (n=448) *vs.* Placebo (n=447)	F (15‐26a)	Dor local, alergia sazonal
Medina et al.^39^ (2010/3)	ECR, MULTI	HPV BV (n=1017) *vs.* Vacina hepatite A (n=1010)	F (10‐14a)	Dor local, eritema, edema, cefaleia, mialgia, fadiga, urticária e síncope
Romanowski et al.^40^ (2011/3)	ECR fase I/II, PC; 2 *vs.* 3 doses de HPV bivalente	2 doses FOR20/20 M0/6 (n=240); 2 doses FOR40/40 M0/6 (n=241); 2 doses FOR40/40 M0/2 (n=240); 3 doses FOR20/20 0/1/6 (n=239)	F (9‐25a)	–
Garland et al.^32^ (2007/5)	ECR, DC, CP, MULTI	HPV QV (n=2673) *vs.* Placebo (n=2672)	F (16‐24a)	Dor local, eritema, edema e prurido local, febre >38,9°C broncoespasmo
Petäjä et al.^41^ (2011/3)	ECR fase III, MULTI	HPV BV (n=616)	F (10‐25a)	–
Esposito et al.^42^ (2011/3)	ECR, esquema padrão 0/1/6 m) *vs.*alternativo (0/1/12 m)	HPV BV Padrão (n=1195) *vs.* HPV BV Alternativo (n=1188)	F (15‐25a)	–
Kang et al.^43^ (2008/5)	ECR, DC, CP	HPV QV (n=117) *vs.* Placebo (n=59)	F (9‐ 23a)	Dor local, eritema, edema e febre
Li et al.^44^ (2011/3)	ECR, DC, CP	HPV QV (n=302) *vs.* Placebo (n=298)	M (9‐15a) e F (9‐45a)	Dor local, edema e prurido

ECR, ensaio clínico randomizado; DC, duplo cego; PC, parcialmente cego; CP, controlado por placebo; MULTI, multicêntrico; HPV, papillomavírus humano; M, masculino; F, feminino; FOR, formulação; m, mês; BV, bivalente; QV, quadrivalente; a, anos.

Entre os estudos analisados, houve apenas um caso de efeito adverso grave relacionado à vacina, broncoespasmo.^32^Nos demais, não houve relatos de efeitos adversos graves ou óbitos relacionados à vacina. A incidência de efeitos adversos foi maior após a primeira dose do esquema e sua ocorrência se reduziu nas doses subsequentes.^23^^,^ 33 ^-^ 36 ^,^ 43

A seleção dos desfechos para as metanálises foi feita de acordo com a frequência de aparecimento dos efeitos adversos estudados nas publicações selecionadas, comparou-se sua ocorrência entre indivíduos vacinados e não vacinados contra o HPV e destacaram-se os efeitos locais (dor, eritema e edema) e, como efeito sistêmico, a febre.

Essa análise foi feita com cinco trabalhos com escore Jadad^31^ igual a cinco,^32^^-^ 34 ^,^ 36 ^,^ 43 sem restrição de sexo ou faixa etária, para vacina quadrivalente. Quatro deles foram multicêntricos^32^^-^ 34 ^,^ 36 e um foi feito na Coreia do Sul.^43^ Os resultados obtidos a partir das metanálises dos desfechos "dor", "edema", "eritema" e "febre" estão demonstrados na Figura 2.

**Figura 2:** Metanálise dos desfechos "dor", "edema", "eritema" e "febre" dos resultados fornecidos pelos ERCs selecionados. *M-H, Mantel-Haenszel; IC, intervalo de confiança. Nos gráficos de floresta, o eixo horizontal representa o IC da diferença de risco; os pontos representam a diferença de risco de cada estudo; os pontos localizados à direita da linha mediana indicam maior incidência do desfecho no grupo que recebeu a vacina; o tamanho dos pontos representa o peso relativo de cada estudo no resultado final; o diamante indica o resultado final da metanálise; **Os gráficos de funil (eixo horizontal=magnitude do efeito; eixo vertical=tamanho da amostra) ilustram a heterogeneidade entre os estudos.

Para o desfecho "dor", a metanálise reuniu cinco ECRs, que totalizaram 12.060 participantes. Desses, 6.348 vacinados para o HPV e 5.712 controlados com placebo. Daqueles vacinados, 4.704 apresentaram dor no local da aplicação, enquanto apenas 3.639 relataram o mesmo desfecho com o uso do placebo, o que resultou em DR=11% (IC95%, 9%-13%; I^2^=93%). Foi, portanto, significativamente mais comum sua ocorrência na aplicação da vacina (*p*<0,001).

O desfecho "edema" foi analisado em três artigos multicêntricos.^32^^,^ 34 ^,^ 36Esses somaram 5.783 pacientes, dos quais 1.154 evoluíram com edema no local de injeção no grupo vacinado para o HPV, enquanto 645 de 5.206 no grupo placebo, que resultou em DR=8% (IC95%, 6%-9%; I^2^=96%) para esse desfecho a favor do grupo vacinado (*p*<0,001).

A metanálise do desfecho "eritema" incluiu os mesmos artigos usados para edema. Dos pacientes vacinados para o HPV, 1.200 desenvolveram eritema no local de aplicação, enquanto 802 apresentaram eritema no grupo placebo, com DR=5% (IC95%, 4%-7%, *p*<0,001, I^2^=88%).

Por sua vez, a metanálise do desfecho "febre" envolveu quatro artigos. Dos 5.900 pacientes vacinados, 609 evoluíram com febre, enquanto 468 dos 5.265 do grupo placebo tiveram o mesmo desfecho. Obteve-se DR=2% (IC95%, 1%-3%, I^2^=64%). Esse resultado foi significativo (*p*<0,003).

## Discussão

Como se trata de uma vacina incorporada recentemente pelo Ministério da Saúde do Brasil ao calendário vacinal de adolescentes do sexo feminino e, ainda, com possibilidade futura de expandir seu público alvo, é imprescindível o estudo do perfil de segurança da vacina papillomavirus humano 6, 11, 16 e 18 (recombinante). Além disso, considerando a sua boa eficácia demonstrada em diversos estudos,^32^^,^ 33 ^,^ 39 ^,^ 41 a relação clara existente entre o câncer de colo de útero e a infecção crônica pelo HPV, a necessidade de mais de uma dose para cumprir seu propósito e o conhecimento das possíveis reações adversas, é importante para garantir a adesão ao esquema proposto e, consequentemente, seu sucesso como estratégia de prevenção dessa doença infecciosa e do câncer de colo uterino.

Nesse contexto, os ensaios clínicos selecionados no presente estudo sugerem a existência de inúmeros efeitos adversos locais e sistêmicos, graves ou não, doenças crônicas de aparecimento no período de estudo e novas condições médicas relevantes relatadas pelos pacientes envolvidos. No entanto, a maioria desses fenômenos clínicos não pode ser definida como efeitos adversos associados à aplicação da vacina papillomavirus humano 6, 11, 16 e 18 (recombinante), pois suas ocorrências não foram comparadas com grupos controle e a relação de causalidade não foi estabelecida.

Em dois artigos, foram testados e comparados esquemas diferentes de vacinação. Não foram encontradas diferenças relevantes na ocorrência de dor ou demais efeitos adversos entre os grupos.^40^^,^ 42 Os 12 artigos restantes fizeram comparação entre grupo submetido a vacina papillomavirus humano 6, 11, 16 e 18 (recombinante) e grupo controle. Para esses 12 trabalhos, os efeitos adversos locais merecem destaque, pois foram os mais frequentemente associados à vacinação contra o HPV, quando comparados com os grupos controle. Em todos esses artigos há pelo menos um efeito adverso estatisticamente associado à vacinação, o que mostra a importância dessa categoria de desfechos. Entre eles estão: dor, eritema, edema e prurido.

A dor foi identificada por 11 dos 12 artigos. Esse foi o efeito adverso mais encontrado, sempre associado à vacina papillomavirus humano 6, 11, 16 e 18 (recombinante) quando comparado com o grupo placebo,^32^^-^ 36 ^,^ 43 ^,^ 44 com o controle com vacina da hepatite A^23^^,^ 37 ^,^ 39 ou com a vacina da hepatite B.^38^ O edema esteve presente em dez artigos. Desses, oito identificaram o edema como efeito diretamente relacionado à vacinação contra o HPV. O eritema foi descrito em nove artigos, mas apenas seis deles demonstraram haver relação direta com a vacinação. O prurido foi efeito adverso pouco encontrado entre os artigos analisados e esteve presente em apenas três.

Dada a magnitude dos efeitos adversos locais identificados, a metanálise objetivou não apenas confirmá-los, mas também quantificá-los por meio de métodos estatísticos que permitam uma avaliação conjunta dos ensaios que investigaram a associação de efeitos adversos com a vacina papillomavirus humano 6, 11, 16 e 18 (recombinante). As metanálises feitas no presente estudo demonstraram maior probabilidade dos indivíduos vacinados desenvolverem efeitos locais com diferença de risco significativa, principalmente no que se refere à dor local e, em menor grau, ao eritema e edema.

Moreira et al.^34^ sugerem que o adjuvante AS04 pode estar implicado na maior ocorrência de efeitos adversos locais, porém o seu papel é de extrema importância para potencializar a imunogenicidade da vacina. Como esse achado foi exclusivo do ensaio feito por esses autores, são necessários mais estudos para determinar essa relação de causalidade, o que pode vir a mudar o uso desse adjuvante na confecção da vacina.

Entre a grande variedade de efeitos adversos sistêmicos identificados nesta revisão, poucos foram de fato relacionados à vacinação, como febre e mialgia. Dez dos 12 artigos listaram a febre como efeito adverso, mas apenas dois deles se relacionaram à vacinação quando comparada com os controles. O risco de febre após a aplicação da vacina papillomavirus humano 6, 11, 16 e 18 (recombinante), evidenciado na metanálise, foi maior do que no grupo placebo. Tal fato deve servir de alerta aos profissionais de saúde que recomendam cuidados após a prescrição da vacina a seus pacientes. No entanto, cabe ressaltar que, apesar de essa diferença de risco ser significativa, não é suficiente para contraindicar o seu uso baseado no benefício de proteção contra a infecção viral e câncer de colo uterino.

Outros efeitos sistêmicos citados, como cefaleia, *rash* cutâneo, mialgia, urticária, síncope, fadiga^39^ e alergia,^33^ foram efeitos adversos sistêmicos também relacionados à vacinação, porém não constaram da metanálise, pois não foram reproduzidos em outros estudos. Os demais efeitos adversos sistêmicos, que divergiram entre os trabalhos, não foram relacionados à vacinação, não apresentaram diferença entre os grupos de estudo na sua ocorrência e colaboraram positivamente para seu perfil de segurança. Alguns deles não tiveram sua frequência de aparecimento explícita, o que dificultou a análise. Tal constatação leva a considerar a necessidade de estudos adicionais em longo prazo e com maior população para que seja possível avaliar desfechos raros.^32^^,^ 39

Apesar de frequentes, os efeitos adversos relacionados à vacinação não foram descritos com características de maior gravidade e em sua grande maioria não foram causa de perdas no cumprimento do esquema vacinal múltiplo. Foi encontrado ainda que a incidência de efeitos adversos é maior após a primeira dose do esquema e sua ocorrência se reduz nas doses subsequentes.^23^^,^ 34 ^-^ 36 ^,^ 43

Nesse contexto, a se considerar a prevalência da infecção pelo HPV na adolescência, a incidência e a morbimortalidade do câncer de colo de útero, a relação risco/benefício da vacina revela-se completamente aceitável e corrobora a sua boa tolerabilidade e segurança proposta por outros autores,^23^^,^ 32 ^-^ 36 ^,^ 39 ^-^ 44 o que contribui para a eficácia das políticas públicas de saúde.

Apesar da identificação de efeitos em estudos observacionais, os quais podem contribuir para a construção do conhecimento, o presente estudo optou pela seleção exclusiva de ECRs com comparação com o grupo controle, principalmente com placebo, pois estudos com esse desenho permitem estabelecer com clareza a associação de efeitos adversos com a vacinação e afastar o efeito indesejável do confundimento, frequentemente presente nos delineamentos observacionais. Assim, os achados aqui encontrados são resultado do conhecimento gerado pelo nível máximo de evidência científica disponível na literatura. Entretanto, cabe ressaltar que os ECRs que avaliam a segurança e reatogenicidade da vacina englobam um número limitado de sujeitos, quando comparados com a população geral de indivíduos elegíveis para vacinação. Essa característica restringe a identificação de efeitos adversos raros ou desconhecidos. Por isso, os resultados aqui apresentados devem ser interpretados com cuidado.

Este estudo não fez distinção entre sexos, pois apesar de a vacinação em massa ter sido recomendada para adolescentes do sexo feminino, a população de homens também é suscetível à infecção e às doenças causadas pelo HPV. Além disso, o sexo masculino pode exercer papel fundamental na transmissão da doença. Deve-se considerar a hipótese de ser um possível grupo das campanhas de vacinação no futuro. Reisinger et al.^36^ sugerem que a população feminina refere mais efeitos adversos do que a população masculina, mas não foi feita comparação formal entre sexos para esse achado. Além disso, os artigos selecionados tiveram grande variabilidade na faixa etária estudada, incluindo escolares, adolescentes, adultos jovens e adultos.

Esse fato contribuiu para diferenças entre as populações de estudo e, possivelmente, para validade externa dos resultados obtidos. Dessa forma, a seleção dos artigos não pôde limitar a faixa etária, pois não havia padrão metodológico entre os estudos quanto à idade da população estudada.

Nas metanálises, foram encontradas valores de heterogeneidade moderada e alta (I^2^>50%), o que diminui o grau de confiança dos resultados aqui apresentados. No geral, os resultados seguiram a tendência do estudo de maior população amostral.^32^ Algumas variáveis podem ser atribuídas como causa da heterogeneidade. Destacam-se a faixa etária, o sexo e o tamanho amostral. Isso sugere que os desfechos estudados nas metanálises podem não ser dependentes somente da vacina, mas também desses fatores citados. Por outro lado, os intervalos de confianças das diferenças de risco calculadas mostraram-se estreitos devido à semelhança dos resultados identificados em cada ensaio selecionado para compor as metanálises, o que reforça a plausibilidade da magnitude de efeito encontrada.

Outra divergência metodológica ocorreu quanto aos grupos controles de cada estudo. Alguns estudos fizeram comparação com grupo controle que usava placebo^32^^-^ 36 ^,^ 43 ^,^ 44 ou controle com vacina da hepatite A^23^^,^ 37 ^,^ 39 ou com vacina da hepatite B.^38^ Esses artigos foram mantidos no presente estudo, pois as vacinas da hepatite A e B já têm seu uso estabelecido, com perfil de segurança bem definido, o que pode ser uma boa referência. Entretanto, a metanálise usou apenas artigos que compararam a vacina papillomavirus humano 6, 11, 16 e 18 (recombinante) com placebo para que a amostra fosse a mais homogênea possível. Ainda, a informação da intensidade dos efeitos adversos estudados não foi demonstrada em todos os trabalhos selecionados, o que pode prejudicar a avaliação da gravidade do efeito. Nosso estudo, portanto, não considerou diferenças de gravidade, quando analisou conjuntamente todos os desfechos.

Embora não tenha sido o foco do presente estudo, é importante ressaltar que quando comparadas às vacinas bivalentes e quadrivalente, os resultados sugerem que ambas apresentam perfil de segurança e efeitos adversos semelhantes, com predominância dos efeitos locais. Porém, esse achado só poderá ser comprovado com estudos adicionais, cujo objetivo seja comparar a ocorrência de efeitos adversos entre as vacinas.

Nesse contexto, os resultados aqui apresentados sugerem que o uso da vacina papillomavirus humano 6, 11, 16 e 18 (recombinante) é potencialmente seguro e bem tolerado. Os principais efeitos adversos relacionados à vacinação são os efeitos locais, como dor, eritema e edema. Como efeito sistêmico, a febre se mostrou associada à vacinação. Ambos os grupos de efeitos adversos não foram considerados graves. Finalmente, conclui-se que a alta imunogenicidade e o perfil de segurança da vacina papillomavirus humano 6, 11, 16 e 18 (recombinante) determinam que seu uso apresente relação risco/benefício vantajosa e favorável como estratégia de prevenção dessa infecção viral e do câncer de colo uterino, o que suscita o persistente encorajamento, por parte dos profissionais de saúde, da prática da vacinação contra o HPV na população de risco.
